# Why Most Psychologists Should Assess and Report Personality

**DOI:** 10.3389/fpsyg.2019.01982

**Published:** 2019-08-28

**Authors:** Adelson Pinon

**Affiliations:** Independent Researcher, Santiago de Compostela, Spain

**Keywords:** personality assessment, reporting standards, methodology, research participants, knowledge accumulation

## Introduction

The person-situation debate is probably one of the most significant debates in the history of psychology since the late 1960s. Most of the empirical issues that ignited the debate have been resolved (e.g., Fleeson, 2004; Mischel and Shoda, 1998; Lucas and Donnellan, 2009). Many researchers recognize that both the characteristics of persons and situations have important effects on behavior: (a) personality traits are useful for predicting cognitions, emotions, and behaviors across many situations and (b) characteristics of a specific situation are useful in making successful predictions of a given individual (Sherman et al., 2015).

The evidence for the importance of personality is overwhelming. A number of meta-analyses have found robust statistically significant relations between personality characteristics and a wide variety of variables. For example, conscientiousness shows consistent relations with job proficiency and training proficiency (Barrick and Mount, 1991); neuroticism and extraversion influence job satisfaction (Judge et al., 2002); males are found to be more assertive and have slightly higher self-esteem than females, while females are higher than males in tendermindedness, extraversion, anxiety, and trust (Feingold, 1994); repressive-defensiveness, trust, emotional stability, locus of control-chance, desire for control, hardiness, positive affectivity, private collective self-esteem, and tension are closely associated with subjective well-being (DeNeve and Cooper, 1998); creative people are more open to new experiences, less conventional and less conscientious, more self-accepting, self-confident, hostile, impulsive, driven, ambitious, and dominant (Feist, 1998); academic performance is found to correlate with agreeableness, conscientiousness, and openness (Poropat, 2009); extraversion is consistently related to three dimensions of transformational leadership: idealized influence-inspirational motivation (charisma), intellectual stimulation, and individualized consideration (Bono and Judge, 2004); individuals increase in measures of social dominance, conscientiousness, and emotional stability, especially in young adulthood (Roberts et al., 2006); neuroticism predicts problematic strategies like wishful thinking, withdrawal, and emotion-focused coping but, like extraversion, also predicts support seeking (Connor-Smith and Flachsbart, 2007); individuals with anxiety, depressive, and/or substance abuse disorders tend to be high on neuroticism and low on conscientiousness (Kotov et al., 2010); the dark triad (narcissism, Machiavellianism, and psychopathy) is more prevalent among men than women and is generally associated with various types of negative psychosocial outcomes such as aggression/delinquency, interpersonal difficulties, sex-related issues, and antisocial tactics (Muris et al., 2017); extraversion, neuroticism, and conscientiousness are correlated with physical activity (Rhodes and Smith, 2006); individuals scoring high on openness to experience tend to value novelty (self-direction and stimulation values) and particularly novel ideas and broadmindedness, whereas individuals who score low on openness to experience tend to value tradition, conformity, and security values (Parks-Leduc et al., 2015); neuroticism and consciousness are the strongest correlates of trait emotional intelligence and ability emotional intelligence (van der Linden et al., 2017); the average effect of genetic contributions to individual differences in personality is 40%, while 60% is due to environmental influences (Vukasović and Bratko, 2015).

Researchers interested in exploring this field further, may want to read Theodore Millon's evolutionary theory of personality and psychopathology (Millon, 2011) and the new edition of the Handbook of personality: Theory and Research (Robins and John, 2019).

## Personality is Missing

The American Psychological Association has recently published new reporting standards for quantitative and qualitative research recommending researchers to report participant characteristics that might influence the data collected, such as major demographic characteristics (e.g., age, sex, ethnicity, socioeconomic status) and important topic-specific information (Appelbaum et al., 2018; Levitt et al., 2018), but failed to mention personality explicitly. One might assume that this omission is perhaps a sign that personality type may not be a pertinent criterium to consider, but this assumption is not supported by the accumulated evidence in the field.

The most likely reason for this omission is that the authors focused exclusively on generating ideas to increase methodological transparency and basically didn't consider new ideas in other areas. There is a growing sense of crisis in psychological science (e.g., Maxwell et al., 2015; Tackett et al., 2017). Investigators have concerns over questionable research practices that result in grossly inflated false positive error rates (e.g., John et al., 2012), reproducibility of reported analyses (e.g., Shrout and Rodgers, 2018), and replication of empirical findings (e.g., Rahal and Open Science Collaboration, 2015). Recommendations designed to speed up knowledge construction in psychology have focused on methodological and procedural steps, such as preregistering hypotheses, addressing measurement error, setting aside data for confirmation, justifying restriction on the samples, or collaborating to replicate, among many others [for a large number of these recommendations, see Shrout and Rodgers (2018)]. The APA papers have addressed many of these suggestions.

I'd argue that knowledge construction is not just a matter of transparency and methodological excellence. It's also a matter of integrating disparate findings into a coherent set of propositions with higher explanatory power. Personality is one of the missing links that may allow such integration. By neglecting the importance of personality, we are not learning as much as we might.

Empirical researchers are encouraged to assess and report personality data in their papers. Documenting this type of information may allow for more nuanced interpretations of results. Meta-analyses will be able to find relevant personality effects not encountered in the original papers, and our understanding of human behavior will be remarkably enhanced by integrating disparate results from different fields using personality as a common denominator. Unlike behavior genetics, personality measurement is inexpensive, and some instruments only require a few minutes of administration time.

Studies with enough statistical power may contemplate incorporating personality either as a moderator variable or as a covariate, depending on the research question. Personality is normally considered a latent variable although whether we consider a variable latent or not depends on the definition used (Bollen, 2002). Regardless of statistical power, investigators shouldn't avoid including a proper description (average and SD) of the personality of participants.

## Discussion

Researchers tend to describe situational variables reasonably well in their studies. Most psychology focus on static situations (i.e., situational variables that do not or cannot change). In these cases, situations are normally well-described by simply referring to the experimentally manipulated stimuli. For more complex situations such as dynamic situations (e.g., a person in a party), researchers have recently developed the Situational Eight DIAMONDS Model (e.g., Rauthmann and Sherman, 2016).

The same cannot be said of personality. Why aren't psychologists regularly including measures of personality in most of their studies? The majority are simply following the current conventions of their field. Some investigators may be unaware of the predictive power of personality, while others may not see the relevance of measuring the construct when it's unrelated with the problem under study.

Researchers interested in assessing and reporting personality may be unsure about how to measure it properly due to the number of tools available. The two most widely used and empirically validated instruments are the NEO Inventories and the Big Five Inventory (Costa et al., 2019). Based on John et al. (2008), the following indications are recommended, depending on participant time constraints: When time is not at a problem, participants are well educated and test-savvy, and the research question targets personality specifically, then the full 240-item NEO-PI-3, a revision of the revised NEO Personality Inventory (Costa and McCrae, 1992, 2010) would be most useful. Otherwise, the 60-item Big Five Inventory 2 (BFI-2; Soto and John, 2017a) is a good alternative, taking only about 6 minutes of administration time. The suggested inventories have good psychometric properties and may facilitate the integration of results across multiple studies. There are also two abbreviated versions of the BFI-2 (Soto and John, 2017b): the 30-item BFI-2-S and the 15-item BFI-2-XS. Other commonly used measures include the 300-item International Personality Item Pool-NEO (Goldberg et al., 2006), and the 100-item Revised HEXACO Personality Inventory (HEXACO-PI-R; Ashton and Lee, 2016). The HEXACO-PI-R has six rather than five factors; it adds an Honesty/Humility factor that the five-factor model includes within its broader Agreeableness factor.

Most psychologists recognize that human beings are complex psychological entities, composed of multiple dimensions. To advance more efficiently toward effective knowledge accumulation, a psychology of the twenty-first century needs to bring the person back. Assessing and reporting personality is a big step in that direction.

## Author Contributions

The author confirms being the sole contributor of this work and has approved it for publication.

### Conflict of Interest Statement

The author declares that the research was conducted in the absence of any commercial or financial relationships that could be construed as a potential conflict of interest.

## References

[B1] Appelbaum M. Cooper H. Kline R. B. Mayo-Wilson E. Nezu A. M. Rao S. M. (2018). Journal article reporting standards for quantitative research in psychology: the APA Publications and Communications Board task force report. Am. Psychol. 73, 3–25. 10.1037/amp0000191 29345484

[B2] Ashton M. C. Lee K. (2016). Age trends in HEXICO-PI-R self-reports. J. Res. Personal. 64, 102–111. 10.1016/j.jrp.2016.08.008

[B3] Barrick M. R. Mount M. K. (1991). The big five personality dimensions and job performance: a meta-analysis. Personnel Psychol. 44, 1–26. 10.1111/j.1744-6570.1991.tb00688.x

[B4] Bollen K. A. (2002). Latent variables in psychology and the social sciences. Ann Rev. Psychol. 53, 605–634. 10.1146/annurev.psych.53.100901.135239 11752498

[B5] Bono J. E. Judge T. A. (2004). Personality and transformational and transactional leadership: a meta-analysis. J. Appl. Psychol. 89, 901–910. 10.1037/0021-9010.89.5.901 15506869

[B6] Connor-Smith J. K. Flachsbart C. (2007). Relations between personality and coping: a meta-analysis. J. Personal. Soc. Psychol. 93, 1080–1107. 10.1037/0022-3514.93.6.1080 18072856

[B7] Costa P. T. McCrae R. R. (2010). NEO-PI-3. London: Sigma Assessment Systems.

[B8] Costa P. T. Jr. McCrae R. R. (1992). NEO PI-R Professional Manual. Odessa, FL: Psychological Assessment Resources.

[B9] Costa P. T. Jr. McCrae R. R. Löckenhoff C. E. (2019). Personality across the life span. Ann. Rev. Psychol. 70, 423–448. 10.1146/annurev-psych-010418-103244 30231002

[B10] DeNeve K. M. Cooper H. (1998). The happy personality: a meta-analysis of 137 personality traits and subjective well-being. Psychol. Bull. 124, 197–229. 10.1037/0033-2909.124.2.197 9747186

[B11] Feingold A. (1994). Gender differences in personality: a meta-analysis. Psychol. Bull. 116, 429–456. 10.1037/0033-2909.116.3.429 7809307

[B12] Feist G. J. (1998). A meta-analysis of personality in scientific and artistic creativity. Personal. Soc. Psychol. Rev. 2, 290–309. 10.1207/s15327957pspr0204_5 15647135

[B13] Fleeson W. (2004). Moving personality beyond the person-situation debate: the challenge and the opportunity of within-person variability. Curr. Direct. Psychol. Sci. 13, 83–87. 10.1111/j.0963-7214.2004.00280.x

[B14] Goldberg L. R. Johnson J. A. Eber H. W. Hogan R. Ashton M. C. Cloninger C. R. (2006). The international personality item pool and the future of public-domain personality measures. J. Res. Personal. 40, 84–96. 10.1016/j.jrp.2005.08.007

[B15] John L. K. Loewenstein G. Prelec D. (2012). Measuring the prevalence of questionable research practices with incentives for truth telling. Psychol. Sci. 23, 524–532. 10.1177/0956797611430953 22508865

[B16] John O. P. Naumann L. P. Soto C. J. (2008). Paradigm shift to the integrative Big Five trait taxonomy: history, measurement, and conceptual issues, in Handbook of Personality: Theory and Research, eds John O. P. Robins R. W. Pervin L. A. (New York, NY: The Guilford Press, 114–158.

[B17] Judge T. A. Heller D. Mount M. K. (2002). Five-factor model of personality and job satisfaction: a meta-analysis. J. Appl. Psychol. 87, 530–541. 10.1037/0021-9010.87.3.530 12090610

[B18] Kotov R. Gamez W. Schmidt F. Watson D. (2010). Linking “big” personality traits to anxiety, depressive, and substance use disorders: a meta-analysis. Psychol. Bull. 136, 768–821. 10.1037/a0020327 20804236

[B19] Levitt H. M. Bamberg M. Creswell J. W. Frost D. M. Josselson R. Suárez-Orozco C. (2018). Journal article reporting standards for qualitative primary, qualitative meta-analytic, and mixed methods research in psychology: the APA Publications and Communications Board task force report. Am. Psychol. 73, 26–46. 10.1037/amp0000151 29345485

[B20] Lucas R. E. Donnellan M. B. (2009). If the person-situation debate is really over, why does it still generate so much negative affect? J. Res. Personal. 43, 146–149. 10.1016/j.jrp.2009.02.009

[B21] Maxwell S. E. Lau M. Y. Howard G. S. (2015). Is psychology suffering from a replication crisis? What does “failure to replicate” really mean? Am. Psychol. 70, 487–498. 10.1037/a0039400 26348332

[B22] Millon T. (2011). Disorders of Personality: Introducing a DSM/ICD Spectrum From Normal to Abnormal (3rd ed.). Hoboken, NJ: John Wiley & Sons Inc.

[B23] Mischel W. Shoda Y. (1998). Reconciling processing dynamics and personality dispositions. Ann. Rev. Psychol. 49, 229–258. 10.1146/annurev.psych.49.1.229 9496625

[B24] Muris P. Merckelbach H. Otgaar H. Meijer E. (2017). The malevolent side of human nature: a meta-analysis and critical review of the literature on the dark triad (narcissism, Machiavellianism, and psychopathy). Perspect. Psychol. Sci. 12, 183–204. 10.1177/1745691616666070 28346115

[B25] Parks-Leduc L. Feldman G. Bardi A. (2015). Personality traits and personal values: a meta-analysis. Personal. Soc. Psychol. Rev. 19, 3–29. 10.1177/1088868314538548 24963077

[B26] Poropat A. E. (2009). A meta-analysis of the five-factor model of personality and academic performance. Psychol. Bull. 135, 322–338. 10.1037/a0014996 19254083

[B27] Rahal R. M. Open Science Collaboration (2015). Estimating the reproducibility of psychological science. Science 349:aac4716 10.1126/science.aac4716 26315443

[B28] Rauthmann J. F. Sherman R. A. (2016). Situation change: Stability and change of situation variables between and within persons. Front. Psychol. 6:1938. 10.3389/fpsyg.2015.01938 26779068 PMC4703053

[B29] Rhodes R. E. Smith N. E. I. (2006). Personality correlates of physical activity: a review and meta-analysis. Br. J. Sports Med. 40, 958–965. 10.1136/bjsm.2006.028860 17124108 PMC2577457

[B30] Roberts B. W. Walton K. E. Viechtbauer W. (2006). Patterns of mean-level change in personality traits across the life course: a meta-analysis of longitudinal studies. Psychol. Bull. 132, 1–25. 10.1037/0033-2909.132.1.1 16435954

[B31] Robins R. W. John O. (2019). Handbook of Personality: Theory and Research (4th ed.). New York, NY: Guilford Press.

[B32] Sherman R. A. Rauthmann J. F. Brown N. A. Serfass D. G. Jones A. B. (2015). The independent effects of personality and situations on real-time expressions of behavior and emotion. J. Personal. Soc. Psychol. 109, 872–888. 10.1037/pspp0000036 25915131

[B33] Shrout P. E. Rodgers J. L. (2018). Psychology, science, and knowledge construction: broadening perspectives from the replication crisis. Ann. Rev. Psychol. 69, 487–510. 10.1146/annurev-psych-122216-011845 29300688

[B34] Soto C. J. John O. P. (2017a). The next Big Five Inventory (BFI-2): developing and assessing a hierarchical model with 15 facets to enhance bandwidth, fidelity, and predictive power. J. Personal. Soc. Psychol. 113, 117–143. 10.1037/pspp0000096 27055049

[B35] Soto C. J. John O. P. (2017b). Short and extra-short forms of the Big Five Inventory−2: the BFI-2-S and BFI-2-XS. J. Res. Personal. 68, 69–81. 10.1016/j.jrp.2017.02.004

[B36] Tackett J. L. Lilienfeld S. O. Patrick C. J. Johnson S. L. Krueger R. F. Miller J. D. Shrout P. E.. (2017). It's time to broaden the replicability conversation: thoughts for and from clinical psychological science. Perspect. Psychol. Sci. 12, 742–756. 10.1177/1745691617690042 28972844

[B37] van der Linden D. Pekaar K. A. Bakker A. B. Schermer J. A. Vernon P. A. Dunkel C. S.. (2017). Overlap between the general factor of personality and emotional intelligence: a meta-analysis. Psychol. Bull. 143, 36–52. 10.1037/bul0000078 27841449

[B38] Vukasović T. Bratko D. (2015). Heritability of personality: a meta-analysis of behavior genetic studies. Psychol. Bull. 141, 769–785. 10.1037/bul0000017 25961374

